# Soursop leaf extract and fractions protects against L-NAME-induced hypertension and hyperlipidemia

**DOI:** 10.3389/fnut.2024.1437101

**Published:** 2024-08-07

**Authors:** Okim Okim Nsor, Babatunde Adebola Alabi, Joseph Ayo Badejo, Faith Afolabi, Okot-Asi Nku-Ekpang, Ezekiel Olugbenga Iwalewa

**Affiliations:** ^1^Neuropharmacology Unit, Department of Pharmacology and Therapeutics, Faculty of Basic Medical Sciences, College of Medicine, University of Ibadan, Ibadan, Nigeria; ^2^Department of Pharmacology and Therapeutics, Faculty of Basic Clinical Sciences, Bowen University, Iwo, Nigeria; ^3^Department of Pharmacology and Therapeutics, Faculty of Medicine and Pharmacy, Kampala International University in Tanzania, Dar es Salaam, Tanzania; ^4^Department of Physiology, Faculty of Basic Medical Sciences, University of Calabar, Calabar, Nigeria

**Keywords:** *Annona muricata* (Annonaceae), N-nitro-L-arginine-methyl ester, blood pressure, captopril, antioxidants

## Abstract

**Introduction:**

Despite the high phenolic content of *Annona muricata*, little is known about its anti-hypertensive and antihyperlipidemic properties. This study evaluated the anti-hypertensive and antihyperlipidemic potential of *A. muricata* leaf extracts.

**Materials and methods:**

Forty-two male Wistar rats were divided into seven groups of six animals each. N-nitro-L-arginine methyl ester (L-NAME) was used to induce hypertension and hyperlipidemia.

**Results:**

Phytochemical screening of *Annona muricata* leaf extracts (AMLE) revealed the presence of saponins, alkaloids, flavonoids, tannins, coumarins, steroids, terpenoids, and phenols. Comparing the methanol extract with the ethyl acetate fraction, quantification revealed that the methanol extract contained more phenolics, flavonoids, and alkaloids. The AMLE rats significantly reduced triglycerides, total cholesterol, LDL, VLDL, atherogenic index, coronary risk index, and blood pressure. The significant decrease in GSH, catalase, SOD, GST, and oxidative stress markers (MDA, nitrites, and MPO) was reversed by AMLE in a dose-dependent manner. Also, the elevated serum levels of TNF-α and IL-1β in the hypertensive rats were attenuated in the treatment groups.

**Discussion:**

This study suggests the potential ameliorative effects of *Annona muricata* leaf extracts against L-NAME-induced hypertension in rats. Notably, the study showed the antioxidant and anti-inflammatory properties of *A. muricata* leaf extracts, which is seen in its ability to attenuate oxidative stress and inflammatory cytokines in L-NAME-induced hypertensive rats. *A. muricata* extracts also decreased atherogenic risk and improved lipid profiles.

## 1 Introduction

Hypertension (HTN) is characterized by systolic blood pressure (SBP) exceeding 140 mmHg or diastolic blood pressure (DBP) exceeding 90 mmHg. It is associated with cardiac hypertrophy as a reaction to elevated blood pressure and volume, and humans become susceptible to other cardiovascular disorders (1). There are regional differences in the prevalence of hypertension; the WHO African Region has been reported with the greatest prevalence (27%), while the WHO American region has the lowest (18%) (2). The current rapid rise in the prevalence of hypertension and other cardiovascular-related disorders in low-income and middle-income countries has been linked to socioeconomic changes and an increase in the acquisition of lifestyle-related risk factors (3).

Clinically, hypertension can be further divided into Primary/Essential and Secondary forms. 95% of cases of HTN are caused by a multifactorial illness called primary HTN. It is frequently linked to obesity, a high family history, and excessive salt intake, which suggests a hereditary susceptibility. However, the remaining 5% is accounted for by secondary hypertension (HTN) (1). Multiple mechanisms, including the sympathetic nervous system, the renin-angiotensin-aldosterone system (RAAS), and endothelial dysfunction, are involved in the pathophysiology of both kinds of hypertension (4, 5). These processes raise peripheral vascular resistance (PVR), cardiac output, and sodium and water retention, which in turn raise blood pressure (6).

Furthermore, the pathogenesis of hypertension has been linked to oxidative stress, which is characterized as an imbalance between oxidants and antioxidants that favors oxidant-induced damage (7). Increased oxidative stress is linked to reactive oxygen species (ROS) and reactive nitrogen species (RNS). This can result in many disorders, such as cancer, diabetes, hypertension, cardiovascular disease, and neurological diseases (8). Inflammation has also been seen to participate in the development of hypertension. Acute-phase proteins like C-reactive protein (CRP) stimulate the release of pro-inflammatory cytokines like interleukin-6 (IL-6), interleukin-1 beta (IL-1β), and tumor necrosis factor-alpha (TNF-α), further promoting inflammation. This is one way that inflammation contributes to the development of hypertension (9).

In treating HTN, effective management of additional cardiovascular risk factors such as lipid disorders, diabetes/glucose intolerance, obesity, and smoking are crucial for the treatment of hypertension. The therapeutic goal of hypertension is less than 140 mmHg for SBP and less than 90 mmHg for DBP (2). To avoid hypertension and its related problems, modifiable risk factors such as poor diets, physical inactivity, alcohol and tobacco use, and obesity should be addressed (10). Antihypertensive drugs such as angiotensin-converting enzyme (ACE) inhibitors, angiotensin II receptor blockers (ARBs), aldosterone antagonists, and direct renin inhibitors have all been reported to possess adverse effects (11). In addition, these drugs cannot manage hypertension along with lipid disorders without a therapeutic combination of antihypertensive and antihypercholesterol agents, thereby increasing the chance of encountering more adverse effects of drugs. The presence of fresh fruits and medicinal plants with abundant antioxidants and phytochemical compounds has been reported to be a safe and better option in the management of coronary heart diseases, hyperlipidemia, and hypertension (12). There are obvious limitations faced with alternative medicinal treatments especially due to limited scientific knowledge on their mechanism of action, however, the urgent demand for available natural products such as phytomedicine is of preference since they are readily available and cheap in underdeveloped and developing countries (13).

*Annona muricata*, the tropical fruit tree also called soursop, is a member of the Annonaceae family. Traditional medicine uses a variety of *A. muricata* parts to treat a wide range of illnesses; the leaves’ decoction is administered topically for its anti-rheumatic and neuralgic properties (14–16). The phenolic compounds found in *A. muricata* are regarded as the most significant phytochemicals because of their water solubility and frequent use as aqueous infusions in conventional medicine (17). Plants contain phenolic chemicals in large quantities, which are essential for defense reactions (18). Hanhineva et al. (19) have shown promise in controlling glucose homeostasis and carbohydrate metabolism, which may lower the risk of metabolic syndrome and its related consequences. Although *A. muricata* is widely used in traditional medicine, there is a paucity of scientific information on its phytochemical components (especially phenol antioxidants) in managing hypertension and lipid disorders. Therefore, we designed this study to evaluate the anti-hypertensive and antihyperlipidemic effects of methanol and aqueous extracts of *A. muricata* leaf in rats.

## 2 Materials and methods

### 2.1 Chemical agents

Methanol (70%), deionized water, acetonitrile, formic acid, absolute ethanol, and hydrochloric acid (37%) were made available from the Ibadan chemical market. Additional compounds utilized include Ellman’s Reagent {5′,5′-Dithiobis (2-nitrobenzoate) DTNB}, Trichloroacetic acid (TCA), Thiobarbituric acid (TBA), and NG-nitro-L-arginine methyl ester (L-NAME; Akscientific, USA). The study additionally made use of interleukin-6, tumor necrosis factor-alpha (TNF-α) ELISA kits (Biolegend, USA), and captopril (25 and 50 mg; PL Holders, Bristol Laboratories Ltd).

### 2.2 Experimental animals

Forty-two healthy adult male Wistar rats (*Rattus norvegicus*) were used for the experiment. The rats were bred in the animal holding Department of Pharmacology and Therapeutics, University of Ibadan. The animals were kept in the experimental animal room for 2 weeks to achieve acclimatization. All animals were kept in a room with a temperature of 28°C ± 5°C and fed standard rat pellets (Growers Feeds, Ibadan, Nigeria). They were also given unlimited access to water *ad libitum*. The animals were exposed to an ambient day-light cycle. The rats were taken care of, following the 3Rs (replacement, reduction, and refinement) of animal experimentation as well as environmental enrichments to minimize discomfort and pain. The study’s animal usage has been approved with ethical approval number UI-ACUREC/072-1222/22.

### 2.3 Sample collection and preparation

Fresh *Annona muricata* leaves were harvested from the Botanical Garden Oyo State, Nigeria. They were identified and verified at the Forestry Research Institute of Nigeria’s Forest Herbarium, Department of Forest Herbarium, in Jericho, Ibadan, Oyo State, Nigeria, where the voucher specimen number FHI 112618 was assigned. The leaves were air-dried at room temperature for four weeks and size-reduced in a Waring blender (Christy and Norris-47362, England) at the Department of Pharmacognosy, University of Ibadan. About 1,000 g of the size-reduced leaves were soaked in 5 litres of 70% methanol and shaked for 72 h at room temperature. The mixture was filtered, and the filtrate evaporated at 60°C using a vacuum rotary evaporator (RE 100B, Bibby Sterilin, United Kingdom). The wet residue was further freeze-dried using a vacuum freeze dryer (FT33-Armfield, England) and was stored until ready to use as a crude extract. About 124.0 g of crude methanol extract was partitioned using n-hexane, ethyl acetate, and water to obtain their respective fractions.

### 2.4 Qualitative phytochemical screening

#### 2.4.1 Alkaloid determination using Harborne’s (1973) method

200 ml of 10% acetic acid in ethanol was poured into a 250 ml beaker containing 5 g of the sample, which was then covered and left to stand for 4 h. After additional filtering, the extract was concentrated to one-quarter of its original volume in a water bath. Subsequently, the extract was supplemented with concentrated ammonium hydroxide dropwise until the precipitate was completely formed. After letting the entire mixture settle, the precipitate was gathered, cleaned with diluted ammonium hydroxide, and filtered. The leftover material, or alkaloid, was then dried and weighed.

#### 2.4.2 Saponin determination

The Saponin determination method applied was the Obadoni and Ochuko method (20). Following the grinding of the samples, 20 g of each was placed in a conical flask along with 100 cm^3^ of 20% aqueous ethanol. Using constant stirring, the samples were heated to approximately 55°C over 4 h in a hot water bath. After filtering the mixture, 200 ml of new 20% ethanol was used to extract the residue once more. At roughly 90°C, the mixed extracts were reduced to 40 ml in a water bath. After transferring the concentrate into a 250 ml separator funnel, 20 ml of diethyl ether was added, and the mixture was violently shaken. The ether layer was disposed of, and the aqueous layer was recovered. There was another purifying procedure. Then n-butanol (60 ml) was added. Twice, 10 milliliters of 5% aqueous were used to wash these mixed n-butanol extracts, sodium chloride, and the remaining solution was heated in a water bath. Following evaporation, the samples were dried in an oven to a set weight, at which point the percentage of saponin was determined.

#### 2.4.3 Determination of total phenolic content

The plant extracts’ phenolic content was ascertained through the application of the spectrophotometric method. Folin–Ciocalteu reagent was used for the determination of the total phenol content (21). The reaction mixture consisting of 1 ml of extract and 9 ml of distilled water was taken in a volumetric flask (25 ml). One milliliter of Folin–Ciocalteu phenol reagent was treated to the mixture and shaken well. After 5 min, 10 ml of 7% Sodium carbonate (Na₂CO_3_) solution was tittered into the mixture. The volume was made up to 25 ml, and a set of standard solutions of gallic acid (20, 40, 60, 80, and 100 μg/ml) were prepared in the same manner as described earlier and incubated for 90 min at room temperature. With a UV/visible spectrophotometer, the absorbance of the test and standard solutions was measured at 550 nm to the reagent blank. The amount of total phenol was stated as mg of GAE/gm of extract.

#### 2.4.4 Determination of tannin content

The tannins were determined by the Folin–Ciocalteu method. About 0.1 ml of the sample extract was added to a volumetric flask (10 ml) containing 7.5 ml of distilled water and 0.5 ml of Folin Ciocalteu Phenol reagent, 1 ml of 35% Na₂CO_3_ solution and dilute to 10 ml with distilled water. The mixture was mixed thoroughly and allowed to settle at room temperature for half an hour. A set of reference standard solutions of gallic acid (20, 40, 60, 80, and 100 μg/ml) were prepared in the same manner as described earlier. Absorbance for test and standard solutions was measured against the blank at 725 nm with a UV/Visible spectrophotometer. The tannin content was expressed in terms of mg of GAE /g of extract (21).

#### 2.4.5 Determination of total flavonoid content

Total flavonoid content was measured by the aluminum chloride colorimetric assay (22). The reaction mixture consisted of 1 ml of extract and 4 ml of distilled water put into a 10 ml volumetric flask. To the flask, 0.30 ml of 5% sodium nitrite was added and after 5 min, 0.3 ml of 10% aluminum chloride was also added. After 5 min, 2 ml of 1 M Sodium hydroxide was treated and diluted to 10 ml with distilled water. Following the previously mentioned procedure, a series of reference standard solutions containing quercetin (20, 40, 60, 80, and 100 μg/ml) were prepared. Using a UV/Visible spectrophotometer, the absorbance of the test and standard solutions was measured at 510 nm to the reagent blank. The whole flavonoid concentration was given in milligrams of QE per gram of extract.

### 2.5 Experimental design

Hypertension was induced in the male Wistar rats weighing (150–200 g) using L-NAME-induced hypertension in rat models as described by Sung and others (23). The animals had fasted for the whole night, but they were allowed unlimited access to drinking water just before the experiment. The rats were randomly assigned to seven groups of six each.

Group 1: In distilled water, the control, non-hypertensive rats received the vehicle dimethyl sulphoxide (DMSO; 10 ml/kg).

Group 2: The hypertensive negative control group; rats received L-NAME (40 mg/kg).

Group 3: The hypertensive rats were treated with a standard ACEI antihypertensive drug (Captopril 40 mg/kg).

Group 4: The hypertensive rats were treated with 50 mg/kg of crude extracts of *A. muricata*.

Group 5: The hypertensive rats were treated with 100 mg/kg of crude extracts of *A. muricata*.

Group 6: The hypertensive rats were treated with 200 mg/kg of crude extracts of *A. muricata*.

Group 7: The hypertensive rats were treated with the aqueous fraction of the crude extracts of *A. muricata* (200 mg/kg).

The animals were treated daily with normal saline, extract, or captopril for 1 h before administering L-NAME for 28 days. The body weights were also measured weekly.

### 2.6 Blood pressure measurement

After treatment, blood pressure (BP) was measured using an electro-sphygmomanometer (CODA, Kent Scientific, USA) in conjunction with a tail-cuff plethysmograph (Hugo-Sachs Elektronik, Freiburg, Germany) that included a warming board to keep the animals warm, occlusion cuffs, volume pressure reading cuffs, and a restraining cone. The animals were acclimated to the process before measuring the systolic, diastolic, and mean arterial pressure. Only during quiescent phases were blood pressure readings taken to reduce stress; analysis was performed using the average measurements. A line graph was used to plot changes in the mean arterial blood pressure, diastolic blood pressure, and systolic blood pressure. The area under the curve was computed to measure total BP changes, and statistical analysis was done to determine significance.

### 2.7 Animal sacrifice and tissue preparation

Following the blood pressure measurement on day 28, the animals were fasted overnight. After that, blood was drawn into plain, anticoagulant-free tubes via the retroorbital venous plexus, and they were put to death using deep anesthesia. After that, the animals were dissected to remove the tissues (kidney, heart, and aorta), and were rinsed in potassium chloride (0.1 M, pH 7.4) and stored at −80°C. To extract the serum, the blood samples were centrifuged for 15 min at 3000 rpm. The serum was then separated into smaller quantities, or aliquots, and kept at −20°C to be analyzed later for pro-inflammatory cytokines and serum nitrite.

The harvested tissues; kidney, heart, and aorta were homogenized in sodium phosphate buffer. (0.1 M, pH 7.4) using a mechanical homogenizer with a glass Teflon. The homogenates were centrifuged at 4°C in a refrigerated centrifuge at a speed of 10, 000 rpm for 10 min to obtain the supernatant.

### 2.8 Assay of serum inflammatory markers and tissue oxidative indices markers

#### 2.8.1 Determination of serum nitrite

Nitrite was measured as an indicator of nitric oxide (NO) production according to the Griess method as described by Misco and other researchers (24). Griess reagent was freshly prepared by mixing equal volumes of 0.1% N-(1-naphthyl) ethylene diamine dihydrochloride and 1% sulphanilamide (in 5% phosphoric acid). After adding 50 μl of the serum to a microtitre plate, it was diluted with 50 μl of distilled water and then incubated for 10 min at room temperature in the dark with 100 μl of Griess reagent. To create the standard curve, sodium nitrite was created as the standard. Using a microplate reader, the absorbance was determined at 540 nm (MICRO READ 1000, Belgium). The sodium nitrite standard curve was used to calculate the nitrite serum concentration, which was then represented as μM nitrite/mg protein.

#### 2.8.2 Enzyme-linked immunosorbent assay for determination of IL-1β and TNF-α

The Biolegend ELISA MAXTM Deluxe kit, USA, was utilized to measure the serum levels of TNF-α and IL-1β. The kit was specific to the cytokines of interest and had a sensitivity limit of 4 pg./ml. Following the guidelines provided by Biolegend, all measurements were carried out at room temperature using a microplate reader equipped with a 450 nm filter. The concentration of IL-1β and TNF-α from the serum was determined by comparing them to the standard curves provided in the ELISA kit and expressed as pg./mg protein (25).

#### 2.8.3 Lipid peroxidation

Tissue malondialdehyde was measured as an index of lipid peroxidation using the assay of thiobarbituric acid reacting substances (TBARs) as described by Nagababu (26). 100 μl of tissue supernatant was diluted 20 times in 0.15 M Tris-KCl buffer, and mixed with 0.5 ml of trichloroacetic acid (30%) and 0.5 ml of TBA (0.75%). Following an hour of heating at 80°C, the mixtures were extracted using 1 ml of butanol. Centrifugation at 3000 g for 5 min was used to separate the organic phase, which was then measured at 532 nm. Next, an index of absorption for MDA (molar extinction coefficient 1.56 × 105 /M/cm) was used to determine the outcome. The TBAR content of the tissues was reported as nmol MDA/g tissue.

#### 2.8.4 Reduced glutathione

Reduced glutathione (GSH) was measured in the aorta, heart, and kidney supernatants using Ellman’s reagent in a method described by Jollow (27). 100 μl of the tissue supernatant was diluted 10 times and deproteinized with 1 ml Trichloroacetic acid (20%). The mixture was centrifuged for 10 min at 10,000 rpm and 4°C. A microplate plate combined 100 μl of 51-Dithios-nitrobenzoic acid (DTNB, 0.0006 M) with 100 μl of the deproteinized supernatant. After that, the absorbance was measured using a microplate reader (MICRO READ 1000, Belgium) at 405 nm in less than 5 min. Using the glutathione standard curve (0–200 μM), the glutathione concentration was calculated and represented as μM GSH/g tissue.

#### 2.8.5 Catalase enzyme assay

The colorimetric assay based on the yellow complex with molybdate and H₂O_2_ was used to measure the amount of catalase present in the tissues of the kidney, heart, and aorta, which was described by Goth and others (28). A microtitre plate was first filled with 50 μl of supernatant, and then with 50 μl of a reaction mixture that contained 65 mmol/ml of H_2_O_2_ in sodium potassium phosphate buffer (60 mM, pH 7.4). After three minutes of incubation, the enzymatic reaction was halted with 100 μl of ammonium molybdate (64.8 mM) in sulfuric acid. The absorbance at 405 nm was measured with a microplate reader (MICRO READ 1000, Belgium). The catalase enzyme activity was measured in units of U/g tissue.

#### 2.8.6 Lipid profile assay

Serum triglycerides, HDL, and total cholesterol were tested using Randox diagnostic kits (Randox Laboratories Limited, Antrim, United Kingdom). Low-density lipoprotein (LDL) was calculated as LDL-Cholesterol = Total cholesterol − [HDL – Cholesterol + (Triglyceride/5)] (29). The Atherogenic index (AI) and Coronary index (CI) were calculated using the formula of Kazemi et al. (30) (AI = LDL-C/HDL-C) and CRI (CRI = TC/HDL-C).

#### 2.8.7 Urea assay

The amount of urea in serum was measured using the ELISA kit, according to manufacturing protocols provided by Randox ELISA kits (Randox Laboratories Limited, Antrim, United Kingdom).

### 2.9 Histopathological analysis of the heart

Formaldehyde with a 10% phosphate buffer was used to fix harvested hearts. The preserved cardiac tissues were processed using a microtome (Leica, Germany) to create tissue blocks embedded in paraffin wax and sectioned in the sagittal plane. To show the general histology of the heart, the cardiac sections were treated via fixation, dehydration, clearing, infiltration, embedding, and staining with hematoxylin and eosin.

### 2.10 Statistical analysis

The Shakiro-Wilk normality test was used for normal distribution. Data are presented as mean ± standard error of mean (SEM) at a 95% confidence interval level and statistical significance was set at *p* < 0.05. Data were analyzed using one-way analysis of variance (ANOVA), and significant differences were further analyzed with Newman-Keul *post hoc* test (multiple comparisons) using GraphPad Prism® software version 5.01 (GraphPad Software, Inc. La Jolla, CA 92037 USA).

## 3 Results

### 3.1 Qualitative and quantitative phytochemical screening

Qualitative and quantitative phytochemical screening is shown in Tables 1–3.

**Table 1:** Qualitative phytochemical screening.

	Parameters	Ethyl acetate	Methanol
1	Saponin (Froth’s Test)	+	+
2	Alkaloid (Hager’s Test)	+	+
3	Flavonoid (Lead acetate test)	+	+
4	Tannin (Braymer’s Test)	+	+
5	Coumarin (Reaction with 10% NaOH)	+	+
6	Steroid (Salkowaski’s Test)	+	+
7	Terpenoid (Salkowaski’s Test)	+	+
8	Cardiac glycosides (Legal’s Test)	+	+
9	Glycosides	+	+
10	Quinones	+	+
11	Anthocyanin	−	−
12	Phytosteroids	+	+
13	Phenols (Ferric chloride test)	+	+

**Table 2:** Quantitative phytochemical: ethyl acetate.

Parameter	1	2	3	Mean	SD
% Saponin	3.82	3.8	3.96	3.86	0.071181
% Alkaloid	8.41	8.64	8.8	8.616667	0.160069
mg/g Flavonoid (QE)	59.777	59.555	60	59.77733	0.181671
mg/g Phenol (GAE)	8.211	8.116	8.11	8.145667	0.046263
mg/g Tannin (GAE)	2.797	2.797	2.766	2.786667	0.014614

**Table 3:** Quantitative phytochemical: methanol.

Parameter	1	2	3	Mean	SD
% Saponin	5.82	4.4	3.96	4.726667	0.793697
% Alkaloid	12.41	12.64	12.2	12.41667	0.179691
mg/g Flavonoid (QE)	71.222	71.555	71.111	71.296	0.188664
mg/g Phenol (GAE)	24.032	23.978	24.005	24.005	0.022045
mg/g Tannin (GAE)	1.411	1.402	1.396	1.403	0.006164

### 3.2 Effect of *A. muricata* treatment on L-NAME-induced hypertension

#### 3.2.1 Effect on blood pressure changes

Compared with normal control and treatment groups, the mean, systolic, and diastolic blood pressure of L-NAME-induced hypertensive rats were significantly high. A significant decrease in systolic blood pressure was observed in the AME 50 mg/kg and AME 200 mg/kg. There was a significant reduction in diastolic blood pressure of AME 200 mg/kg, Captopril 40 mg/kg, and AME-RAF 200 mg/kg. Mean arterial blood pressure shows a significant decrease in AME 50 mg/kg, AME 200 mg/kg, Captopril 40 mg/kg, and AME-RAF 200 mg/kg. Compared to the AME 50 mg/kg and Captopril 40 mg/kg groups, the results show a more notable decrease in the AME 200 mg/kg group (Table 4).

**Table 4:** Effect of *Annona muricata* extracts on the systolic, diastolic, and mean blood pressure of L-NAME-induced hypertensive rats.

Mean blood pressure (mmHg)	Systolic blood pressure (mmHg)	Diastolic blood pressure (mmHg)
Group 1 (Positive Control): 124.715 ± 14.57	154.581 ± 15.29	109.534 ± 15.57
Group 2 (L-NAME Group): 134.994 ± 21.64^*^	169.813 ± 20.45^*^	116.981 ± 24.93^*^
Group 3 (AME 50 mg/kg): 95.229 ± 40.81^#^	116.145 ± 45.71^#^	84.078 ± 40.58
Group 4 (AME 100 mg/kg): 108.913 ± 25.23	142.377 ± 29.49	90.365 ± 27.38
Group 5 (AME 200 mg/kg): 90.850 ± 29.17^#^	133.078 ± 28.11^#^	69.070 ± 32.03^#^
Group 6 (Captopril 40 mg/kg): 115.571 ± 14.42^#^	152.507 ± 14.24	95.353 ± 19.11^#^
Group 7 (AME-RAF 200 mg/kg): 101.297 ± 23.83^#^	142.382 ± 37.45	76.686 ± 26.21^#^

Each bar represents the mean ± S.E.M (*n* = 5). ^#^*p* < 0.05 compared with the control group (One-way ANOVA followed by Newman-Keul *post hoc* test). ^*^p < 0.05 compared with the L-NAME group (One-way ANOVA followed by Newman-Keul *post hoc* test). L-NAME, NG-nitro-L-arginine methyl ester; AME, *Annona muricata* extracts; RAF, Residual aqueous fraction.

#### 3.2.2 Effect on body weight

**Figure 1:**
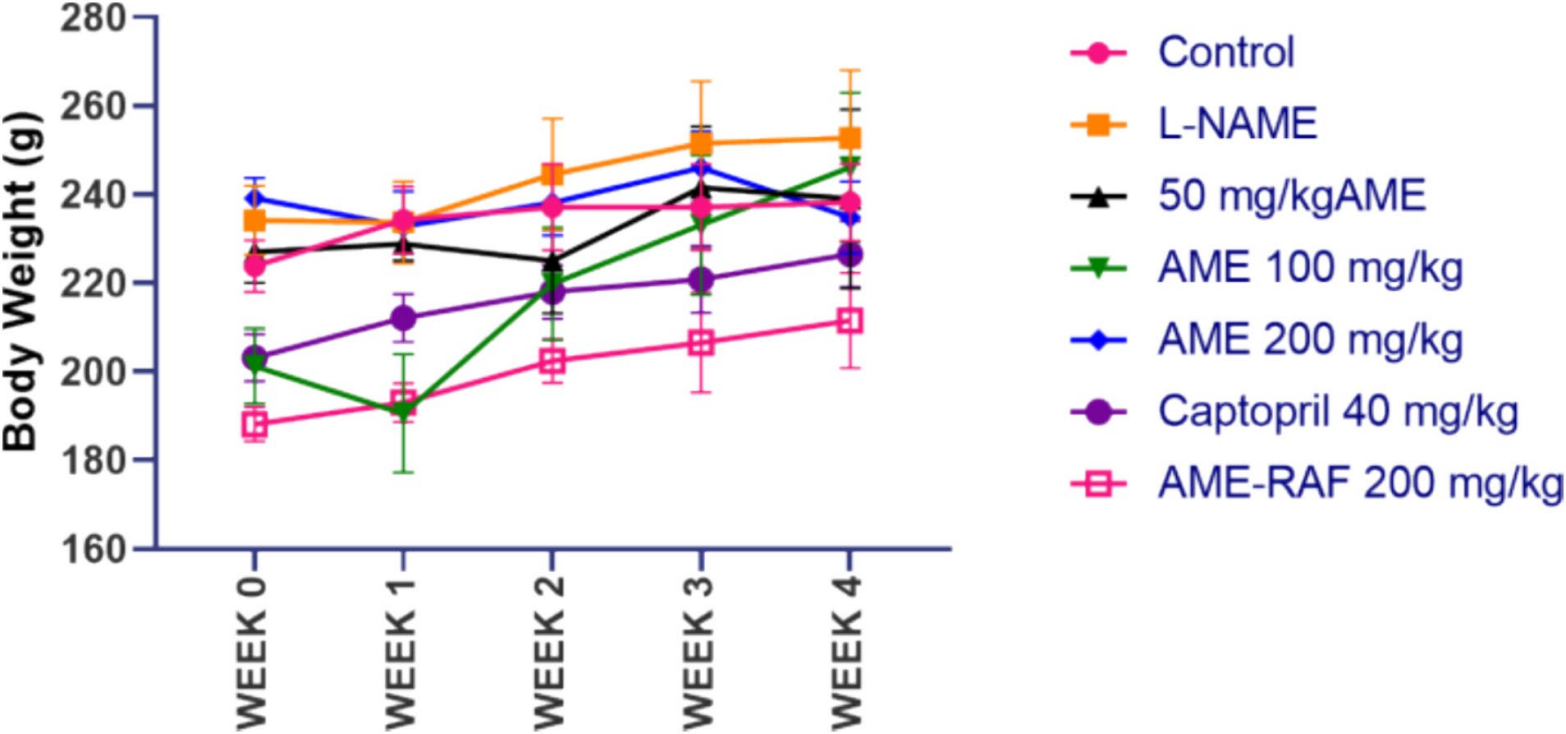
Effect of *A. muricata* extracts on body weight in L-NAME-induced hypertensive rats. Results are expressed as mean ± S.E.M (*n* = 5). ^#^*p* < 0.05 compared with control group, ^*^p < 0.05 compared with L-NAME group.

The effect of hypertension on body weight is shown in Figure 1. Although there was a continuous increase in the body weight of rats throughout 4 weeks, there was no significant difference in the body weight of the animals across the groups.

### 3.3 Effect of *A. muricata* extracts on lipid profile in L-NAME-induced hypertensive rats

#### 3.3.1 Effect of *A. muricata* extracts on triglycerides in L-NAME-induced hypertensive rats

From the results representation of Table 5, the serum triglyceride levels were significantly increased in the L-NAME-induced hypertensive group compared with the rats in the control group. Treatment of rats with AME 200 mg/kg and Captopril 40 mg/kg decreased the triglyceride levels (*p* < 0.05) when compared to the negative L-NAME-induced hypertensive rats. The 50 mg/kg and 100 mg/kg AME did not have any significant effect on the serum triglyceride level. The aqueous fraction at 200 mg could not exert any effect on the serum triglyceride level. There was no significant effect of L-NAME and extract on the total cholesterol level of rats, but the treatment of rats with captopril 40 mg/kg decreased the serum level of total cholesterol level significantly. There was no significant effect of L-NAME-induced hypertension, *A. muricata* extracts, and captopril on the serum levels of high-density lipoprotein in rats. There was a significant increase in the serum LDL-cholesterol levels of the L-NAME hypertensive group compared to the normal control group. The AME-100 mg/kg treated rats were the only treatment group that reduced the serum LDL-cholesterol level significantly. Also, Table 5 shows a significant increase in the serum level of VLDL cholesterol of the L-NAME hypertensive group compared to the control group. AME 200 mg/kg and Captopril 40 mg/kg groups showed a significant (*p* < 0.05) decrease in VLDL cholesterol levels in comparison with the L-NAME hypertensive group. A significant increase (*p* < 0.05) in atherogenic index was observed in the L-NAME hypertensive group compared to the control group. All the treatment groups (AME-50, 100, 200 mg/kg, AME-RAF 200 mg/kg, and Captopril 40 mg/kg) revealed a significant decrease (*p* < 0.05) in atherogenic index. There was no significant difference between the high-density/low-density lipoprotein ratio of the L-NAME hypertensive group and the control group. A significant increase (*p* < 0.05) in the high-density/low-density lipoprotein ratio was observed in the AME (100, 200 mg/kg). The high-density/low-density lipoprotein ratio was significantly reduced (*p* < 0.05) in the AME 50 mg/kg, Captopril, 40 mg/kg, and AME-RAF 200 mg/kg treatment rats. There was a significant increase (*p* < 0.05) in the coronary risk index of the L-NAME-induced hypertensive rats when compared with the control group. A significant decrease (p < 0.05) in the coronary risk index was seen in the treatment groups (AME-50, 100, 200 mg/kg, and Captopril 40 mg/kg) compared with the L-NAME group. However, no significant change was observed in the AME-RAF 200 mg/kg at (*p* < 0.05). Compared with the control group, there was a significant increase (*p* < 0.05) in urea levels of the L-NAME group. However, there was no significant difference (*p* < 0.05) in the urea levels of all treatment groups when compared with the L-NAME and normal control group.

**Table 5:** Effect of *Annona muricata* extracts on lipid profile and renal injury marker of L-NAME induced hypertensive rats.

Lipid profile	UNITS	Group 1 (Positive Control)	Group 2 (L-NAME)	Group 3 (AME 50 mg/kg)	Group 4 (AME 100 mg/kg):	Group 5 (AME 200 mg/kg)	Group 6 (Captopril 40 mg/kg)	Group 7 (AME-RAF 200 mg/kg)
Triglycerides (TG)	(mg/dl)	80.51 ± 4.963	95.91 ± 4.387 ^#^	86.19 ± 4.731	85.99 ± 6.950	81.52 ± 5.360 ^*^	79.56 ± 9.474 ^*^	94.69 ± 6.829
Total Cholesterol (TC)	(mg/dl)	79.28 ± 3.800	83.79 ± 3.730	81.89 ± 4.680	78.83 ± 4.432	84.66 ± 4.692	75.76 ± 4.301 ^*^	91.07 ± 4.815
High-density lipoprotein HDL-C	(mg/dl)	50.56 ± 2.673	48.25 ± 2.779	51.30 ± 2.309	52. 32 ± 3.152	51.56 ± 2.001	46.78 ± 2.417	51.09 ± 3.059
Low-density lipoprotein LDL-C	(mg/dl)	12.56 ± 1.135	16.36 ± 1.681 ^#^	13.71 ± 2.007	7.147 ± 2.166 ^*^	16.79 ± 1.403	13.29 ± 1.909	21.04 ± 1.625 ^*^
Very low-density lipoprotein VLDL-C	(mg/dl)	16.10 ± 1.074	19.18 ± 1.545 ^#^	17.24 ± 1.325	17.20 ± 0.681	16.30 ± 1.072 ^*^	15.91 ± 1.146 ^*^	18.94 ± 1.366
Atherogenic Index (AI)		0.599 ± 0.039	1.010 ± 0.036 ^#^	0.667 ± 0.049 ^*^	0.657 ± 0.068 ^*^	0.621 ± 0.065 ^*^	0.694 ± 0.032 ^*^	0.875 ± 0.067 ^*^
High-density/low-density lipoprotein ratio (HDL/LDL)	(mg/dl)	4.889 ± 0.519	4.718 ± 0.480	0.428 ± 0.208 ^*^	5.632 ± 0.574 ^*^	6.562 ± 0.393 ^*^	1.643 ± 0.538 ^*^	3.264 ± 0.594 ^*^
Coronary risk index (CRI)		1.571 ± 0.066	1.783 ± 0.102 ^#^	1.600 ± 0.096 ^*^	1.532 ± 0.082 ^*^	1.567 ± 0.086 ^*^	1.625 ± 0.052 ^*^	1.808 ± 0.095
UREA	U/L	6.648 ± 0.502	7.912 ± 0.462 ^#^	7.48 ± 0.451	7.681 ± 0.543	7.948 ± 0.335	7.610 ± 0.472	7. 614 ± 0.439

Each bar represents the mean ± S.E.M (n = 5). ^#^*p* < 0.05 compared with the control group (One-way ANOVA followed by Newman-Keul *post hoc* test). ^*^p < 0.05 compared with L-NAME group (One-way ANOVA followed by Newman-Keul *post hoc* test). L-NAME, NG-nitro-L-arginine methyl ester. AME, *Annona muricata* extracts; RAF, Residual aqueous fraction.

### 3.4 Effect of *A. muricata* extract and fraction on oxidative stress and antioxidant parameters of L-NAME-induced hypertensive rats

#### 3.4.1 Effect of *A. muricata* extract and fraction on reduced glutathione of L-NAME-induced hypertensive rats

**Figure 2:**
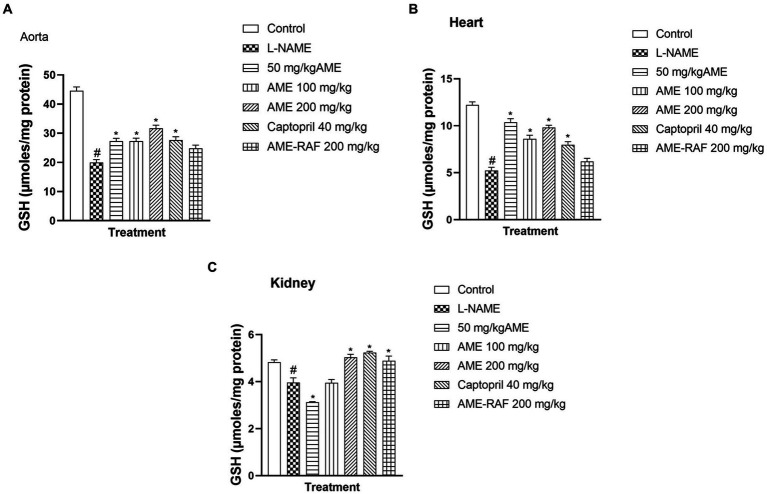
Effect of *A. muricata* extracts on reduced glutathione (GSH) in the aorta, heart and kidney of L-NAME-induced hypertensive rats. Results are expressed as mean ± S.E.M (*n* = 5). ^#^*p* < 0.05 compared with control group, ^*^p < 0.05 compared with L-NAME group.

When compared to the control group, the aortic, myocardial, and renal tissue levels of reduced glutathione (GSH) of the L-Name-induced hypertension group were significantly low (*p* < 0.05). In the aorta of rats, there was a significant increase (*p* < 0.05) in GSH concentration when the L-NAME group was compared with the treatment groups (AME-50, 100, 200 mg/kg, and Captopril 40 mg/kg; Figure 2A). However, there was no significant difference in the AME-RAF 200 mg/kg treatment rats (*p* < 0.05). There was a significant increase (*p* < 0.05) in the cardiac tissue GSH levels of the treatment groups (AME-50, 100, 200 mg/kg, and Captopril 40 mg/kg) compared to the L-NAME group, and the AME-RAF 200 mg/kg treatment rats showed no significant difference (Figure 2B). The renal tissue showed a significant increase (*p* < 0.05) in GSH level when comparing the L-NAME group with the treatment groups (AME-200 mg/kg, Captopril 40 mg/kg, and AME-RAF 200 mg/kg). However, no significant difference was observed in the AME 100 mg/kg group of rats while the GSH levels of AME -50 mg/kg treatment rats decreased further when compared with L-NAME-induced hypertension rats (*p* < 0.05; Figure 2C).

#### 3.4.2 Effect of *A. muricata* extract and fraction on malondialdehyde level of L-NAME-induced hypertensive rats

**Figure 3:**
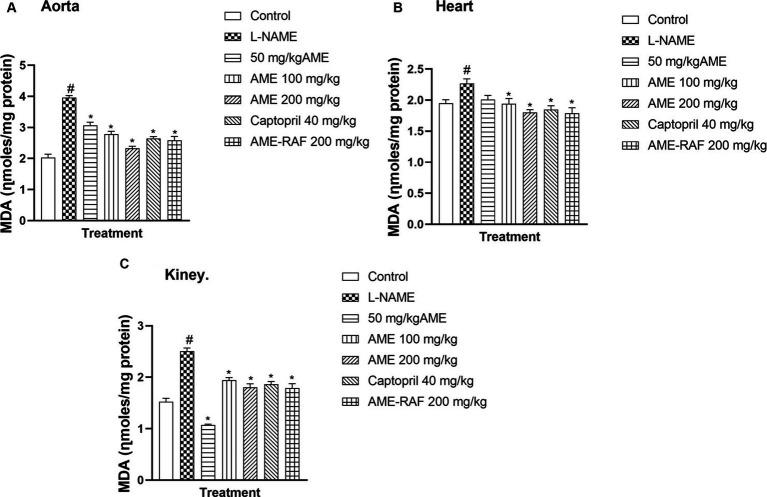
Effect of *Annona muricata* extracts on Malondialdehyde (MDA) in the aorta, heart and kidney of L-NAME-induced hypertensive rats. Results are expressed as mean ± S.E.M (*n* = 5). ^#^*p* < 0.05 compared with control group, ^*^p < 0.05 compared with L-NAME group.

There was a significant increase (*p* < 0.05) in the aortic, myocardial, and renal tissue levels of malondialdehyde (MDA) in the L-NAME-induced hypertension group when compared with the control group (Figures 3A–C). The aortic tissue showed a significant decrease (p < 0.05) in the tissue MDA level of the treated rats (AME-50, 100, 200 mg/kg, Captopril 40 mg/kg, and AME-RAF 200 mg/kg) when compared with the L-NAME group. The heart tissue of treatment rats showed a significant decrease in MDA level (*p* < 0.05) when compared to the L-NAME-induced hypertension rats (AME-100, 200 mg/kg, Captopril 40 mg/kg, and AME-RAF 200 mg/kg). The renal tissue also showed a significant increase (*p* < 0.05) in MDA concentration in all the treatment groups (AME-50, 100, 200 mg/kg, Captopril 40 mg/kg, and AME-RAF 200 mg/kg) when compared with the L-NAME control group.

#### 3.4.3 Effect of *Annona muricata* extract and fraction on nitrites level of L-NAME-induced hypertensive rats

**Figure 4:**
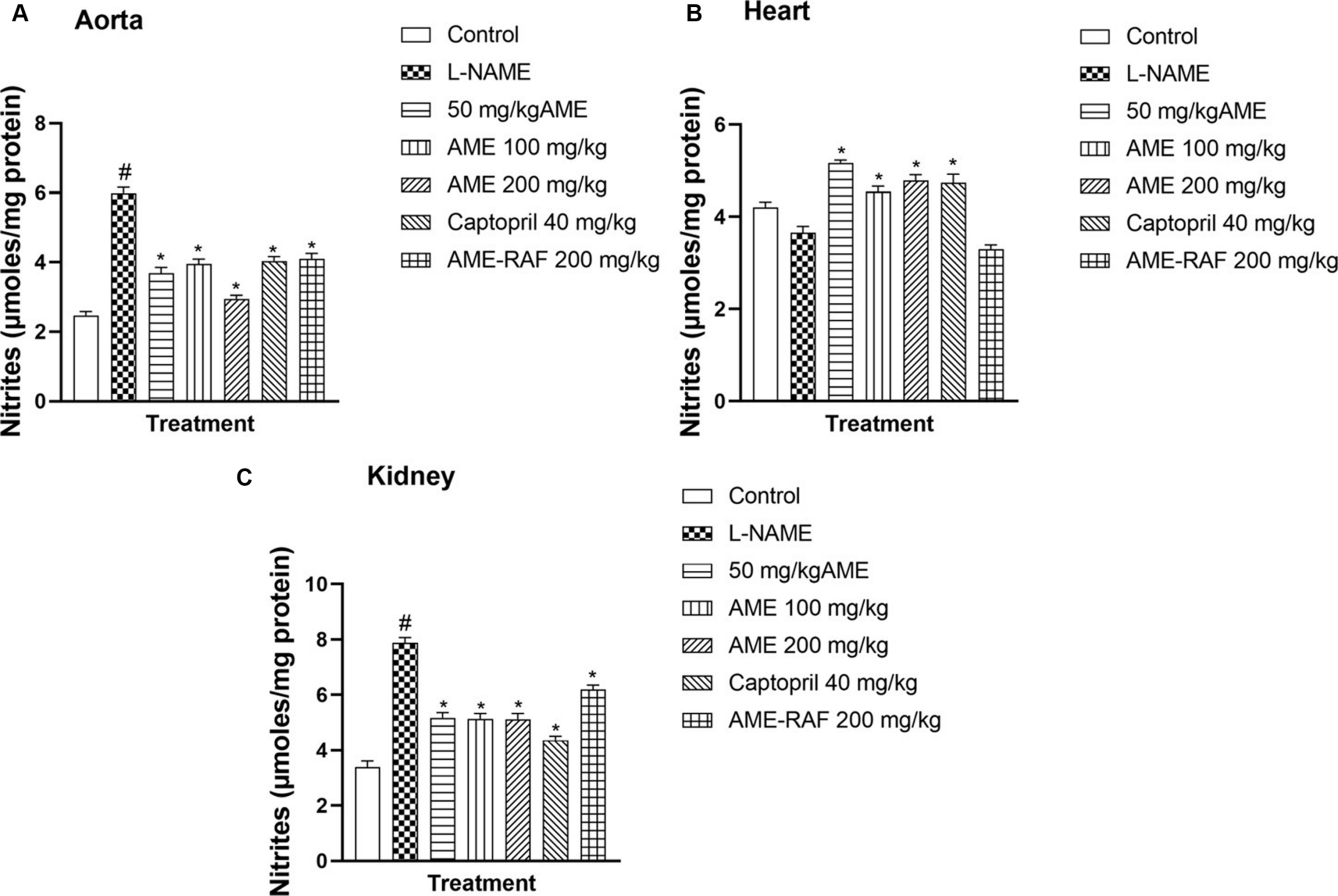
Effect of *Annona muricata* extracts on nitrite concentration in the aorta, heart, and kidney of L-NAME-induced hypertensive rats. Results are expressed as mean ± S.E.M (*n* = 5). ^#^*p* < 0.05 compared with control group, ^*^p < 0.05 compared with L-NAME group.

The aorta and renal tissue of rats in the L-NAME hypertensive group revealed a significantly increased (*p* < 0.05) nitrite level when compared with the normal control group. The cardiac tissue level of nitrite was decreased (*p* > 0.05) when compared with the normal control group (Figures 4A–C). The aortic tissues of treatment rats (AME-50, 100, 200 mg/kg, Captopril 40 mg/kg, and AME-RAF 200 mg/kg) showed a significant decrease (*p* < 0.05) in nitrite level when compared with the L-NAME group. There was a significant increase (*p* < 0.05) in the cardiac nitrite levels of the treatment rats (AME-50, 100, 200 mg/kg, and Captopril 40 mg/kg) when compared with the L-NAME hypertensive rats and no significant difference was seen in the cardiac nitrite level of AME-RAF 200 mg/kg treated rats. The kidney showed a significant decrease (*p* < 0.05) in nitrite in all the treatment groups (AME-50, 100, 200 mg/kg, Captopril 40 mg/kg, and AME-RAF 200 mg/kg) when compared with the L-NAME group.

#### 3.4.4 Effect of *A. muricata* extract and fraction on tissue catalase level of L-NAME-induced hypertensive rats

**Figure 5:**
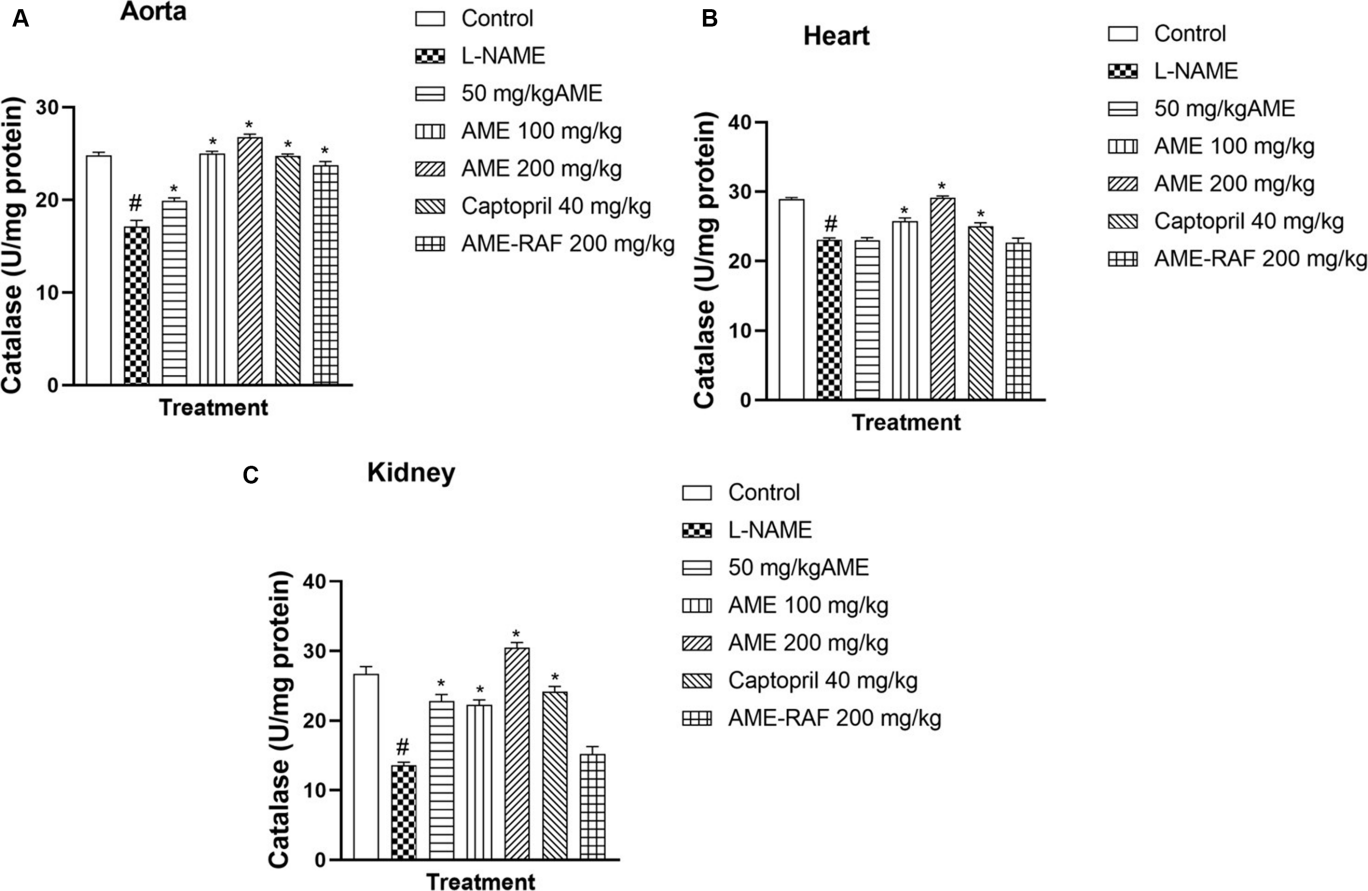
**(A)** Effect of *Annona muricata* extracts on catalase concentration in the aorta, heart and kidney of L-NAME-induced hypertensive rats. Results are expressed as mean ± S.E.M (*n* = 5). ^#^*p* < 0.05 compared with control group, ^*^p < 0.05 compared with L-NAME group.

The aorta, cardiac, and renal tissue of rats in the L-NAME hypertensive group showed that catalase level was significantly reduced (*p* < 0.05) when compared with the normal control group. The aortic tissue catalase levels were increased significantly, (*p* < 0.05) across all the treatment groups (AME-50, 100, 200 mg/kg, Captopril 40 mg/kg, and AME-RAF 200 mg/kg) when compared with the L-NAME group. Similarly, the treatment of rats with AME-100, 200 mg/kg, and Captopril 40 mg/kg, increased the heart tissue level of catalase significantly (*p* < 0.05) when compared with the L-NAME group. Also, the treatment of rats with AME-50, 100, 200 mg/kg, and Captopril 40 mg/kg, increased the renal tissue level of catalase significantly (*p* < 0.05) when compared with the L-NAME control rat (Figures 5A–C).

#### 3.4.5 Effect of *Annona muricata* extract and fraction on superoxide dismutase level of L-NAME-induced hypertensive rats

**Figure 6:**
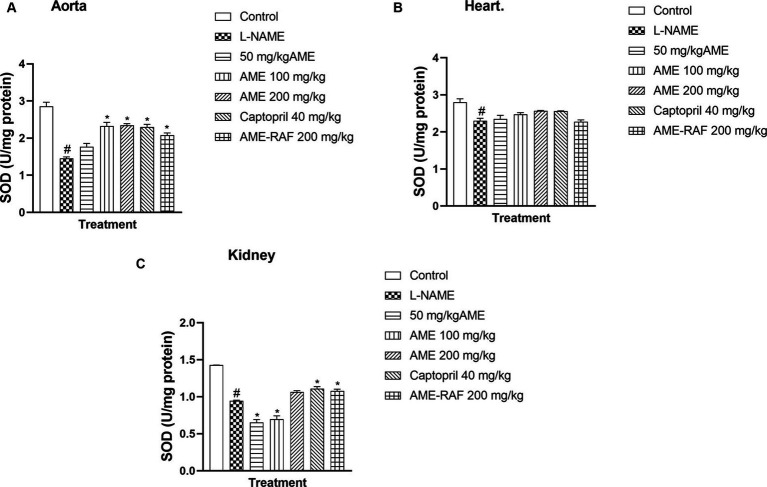
Effect of *Annona muricata* extracts on SOD concentration in the aorta, heart and kidney of L-NAME-induced hypertensive rats. Results are expressed as mean ± S.E.M (*n* = 5). ^#^*p* < 0.05 compared with control group, ^*^p < 0.05 compared with L-NAME group.

L-NAME-induced hypertension suppressed the myocardial, renal, and aortic tissue levels of SOD significantly (*p* < 0.05). Apart from the 50 mg/kg AME treatment group with a non-significant SOD increase, the aortic tissue showed an increase (*p* < 0.05) in SOD concentration in the treatment groups (AME-100, 200 mg/kg, Captopril 40 mg/kg, and AME-RAF 200 mg/kg) when compared with the L-NAME group. The heart tissue showed no significant difference in SOD levels across all treatment groups. The renal tissue showed a significant increase (*p* < 0.05) in SOD concentration in the Captopril 40 mg/kg and AME-RAF 200 mg/kg groups (Figures 6A–C).

#### 3.4.6 Effect of *Annona muricata* extract and fraction on tissue glutathione-S-transferase level of L-NAME-induced hypertensive rats

**Figure 7:**
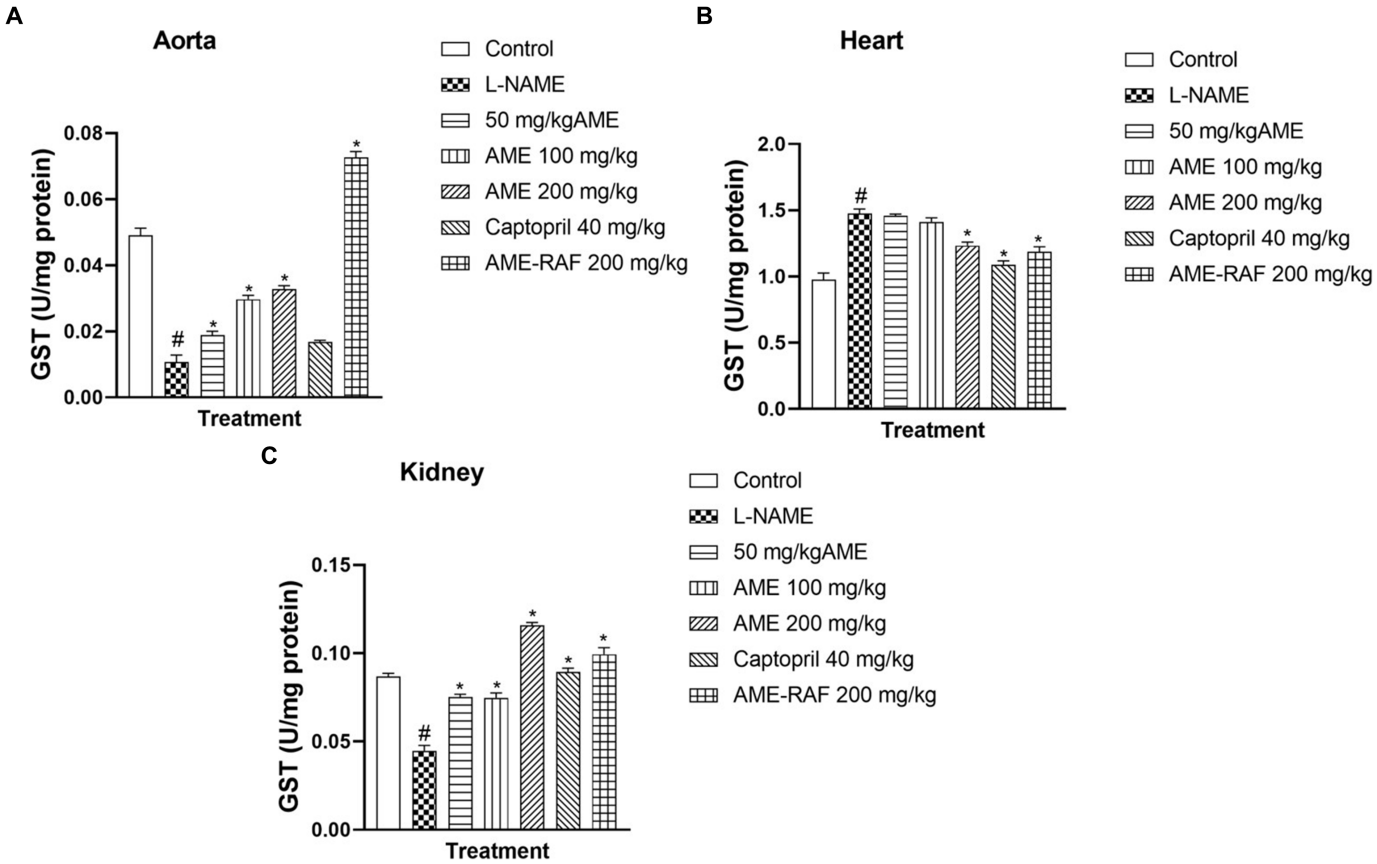
Effect of *Annona muricata* extracts on GST concentration in the aorta, heart and kidney of L-NAME-induced hypertensive rats. Results are expressed as mean ± S.E.M (*n* = 5). ^#^*p* < 0.05 compared with control group, ^*^p < 0.05 compared with L-NAME group.

The aortic and renal tissues showed a significant increase (*p* < 0.05) in the GST levels of the treatment groups (AME-50, 100, 200 mg/kg, and AME-RAF 200 mg/kg) when compared to the L-NAME control group. The heart tissue level of GST was significantly increased (*p* < 0.05) in the L-NAME group when compared with the control group while the treatment groups (AME-200 mg/kg, Captopril 40 mg/kg, and AME-RAF 200 mg/kg) showed a significant decrease in GST level, compared with the L-NAME group (Figure 7).

### 3.5 Effect of *A. muricata* extract and fraction on inflammatory mediators of L-NAME-induced hypertensive rats

#### 3.5.1 Effect of *Annona muricata* extract and fraction on tissue myeloperoxidase level of L-NAME-induced hypertensive rats

**Figure 8:**
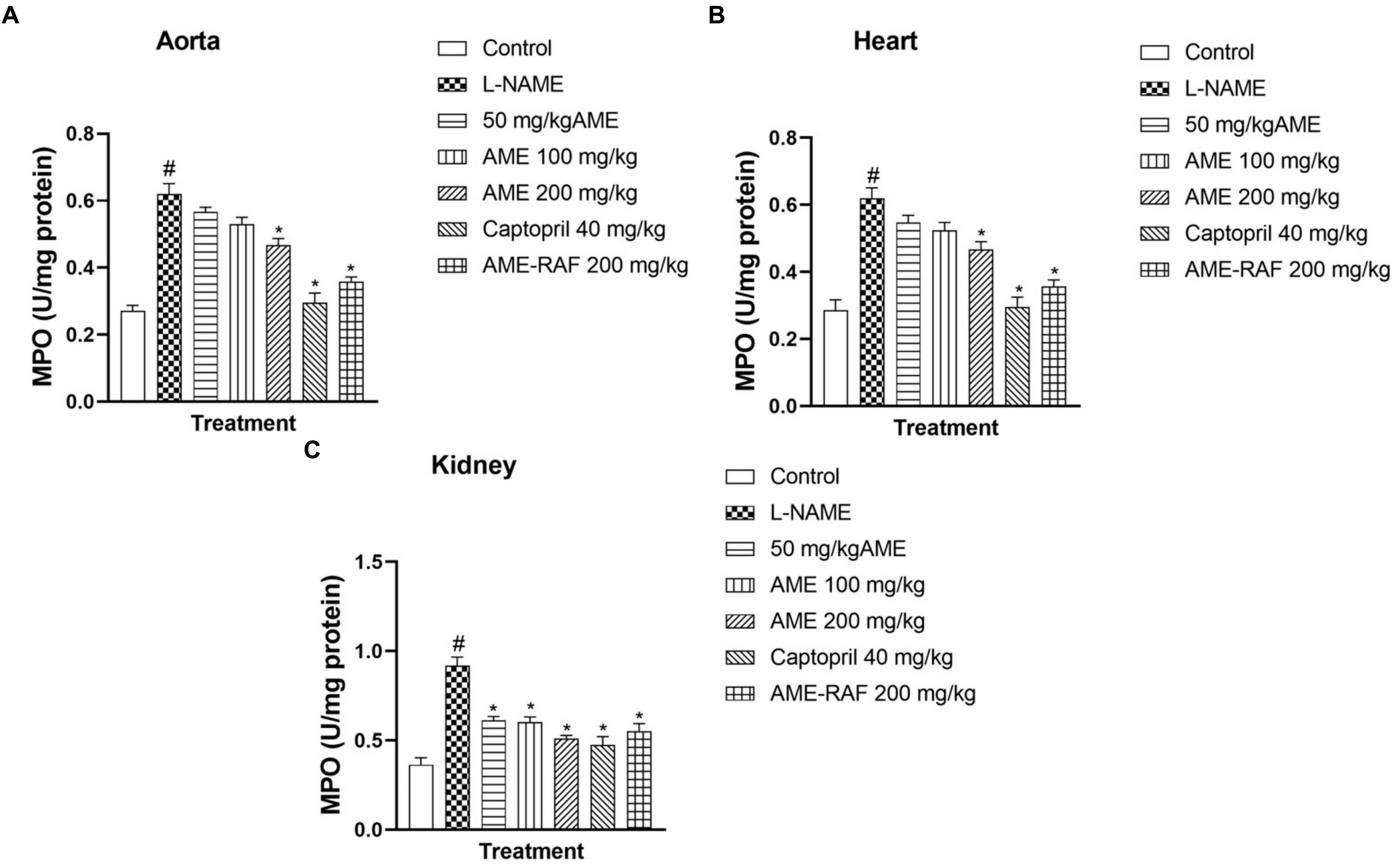
Effect of *Annona muricata* extracts on MPO concentration in the aorta, heart and kidney of L-NAME-induced hypertensive rats. Results are expressed as mean ± S.E.M (*n* = 5). ^#^*p* < 0.05 compared with control group, ^*^p < 0.05 compared with L-NAME group.

L-NAME-induced hypertension significantly enhanced the myocardial, renal, and aortic tissue levels of MPO (*p* < 0.05) compared to normal control rats. The aorta and heart tissue of treated rats (AME-200 mg/kg, Captopril 40 mg/kg, and AME-RAF 200 mg/kg) showed a significant decrease (*p* < 0.05) in MPO concentration when compared with the L-NAME-induced hypertensive rats (Figures 8A,B). The renal tissue showed a significant decrease (*p* < 0.05) in MPO concentration in all the treatment groups (AME-50, 100, 200 mg/kg, Captopril 40 mg/kg, and AME-RAF 200 mg/kg) when compared with the L-NAME-induced hypertensive rats (Figure 8A).

#### 3.5.2 Effect of *Annona muricata* extract and fraction on serum level of tumor necrosis factor-alpha and Interleukin-1 beta in L-NAME-induced hypertensive rats

**Figure 9:**
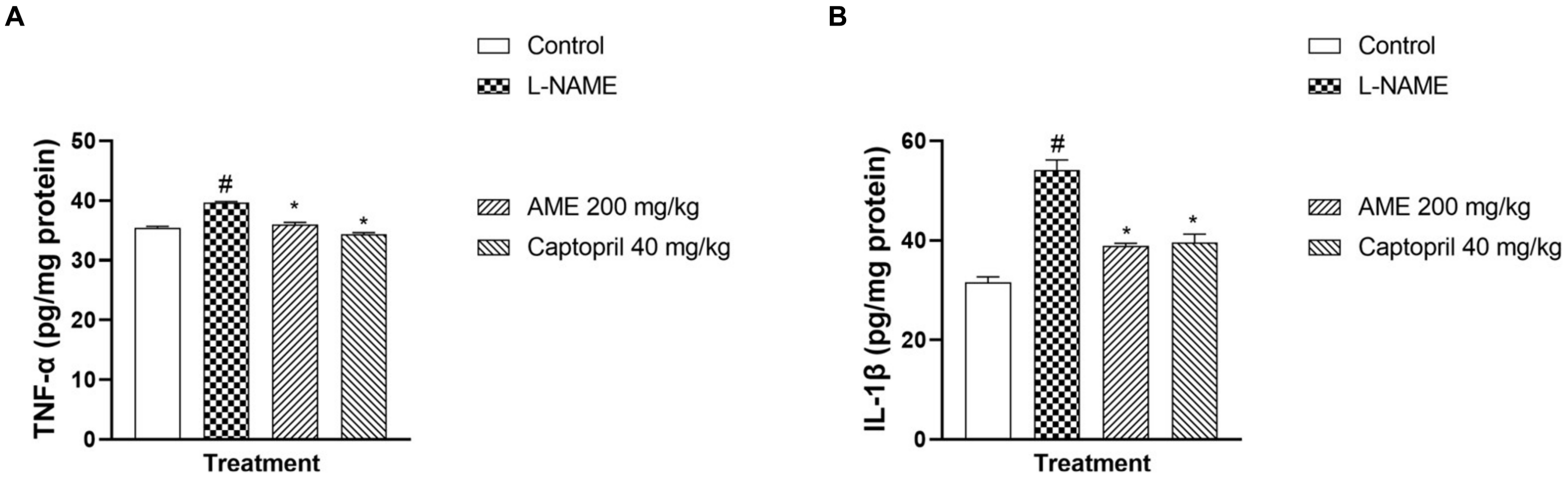
Effect of *Annona muricata* extracts on tumor necrosis factor-alpha and interleukin-1 beta in L-NAME-induced hypertensive rats. Results are expressed as mean ± S.E.M (n = 5). ^#^*p* < 0.05 compared with control group, ^*^p < 0.05 compared with L-NAME group.

Figure 9A shows a significant increase (*p* < 0.05) in tumor necrosis factor-alpha in the L-NAME group compared to the normal control. A significant decrease (*p* < 0.05) in TNF-α was seen in the treatment groups (AME-200 mg/kg, Captopril 40 mg/kg) when compared with the L-NAME group. Figure 9B shows a significant decrease (*p* < 0.05) in the serum level of Interleukin-1 beta (IL-1β) of the L-NAME-induced hypertensive rats. In the treatment groups (AME-200 mg/kg, Captopril 40 mg/kg), there was a significant decrease (*p* < 0.05) in the serum level of IL-1β.

### 3.6 Histological evaluation

#### 3.6.1 Photomicrograph of the aorta

**Figure 10:**
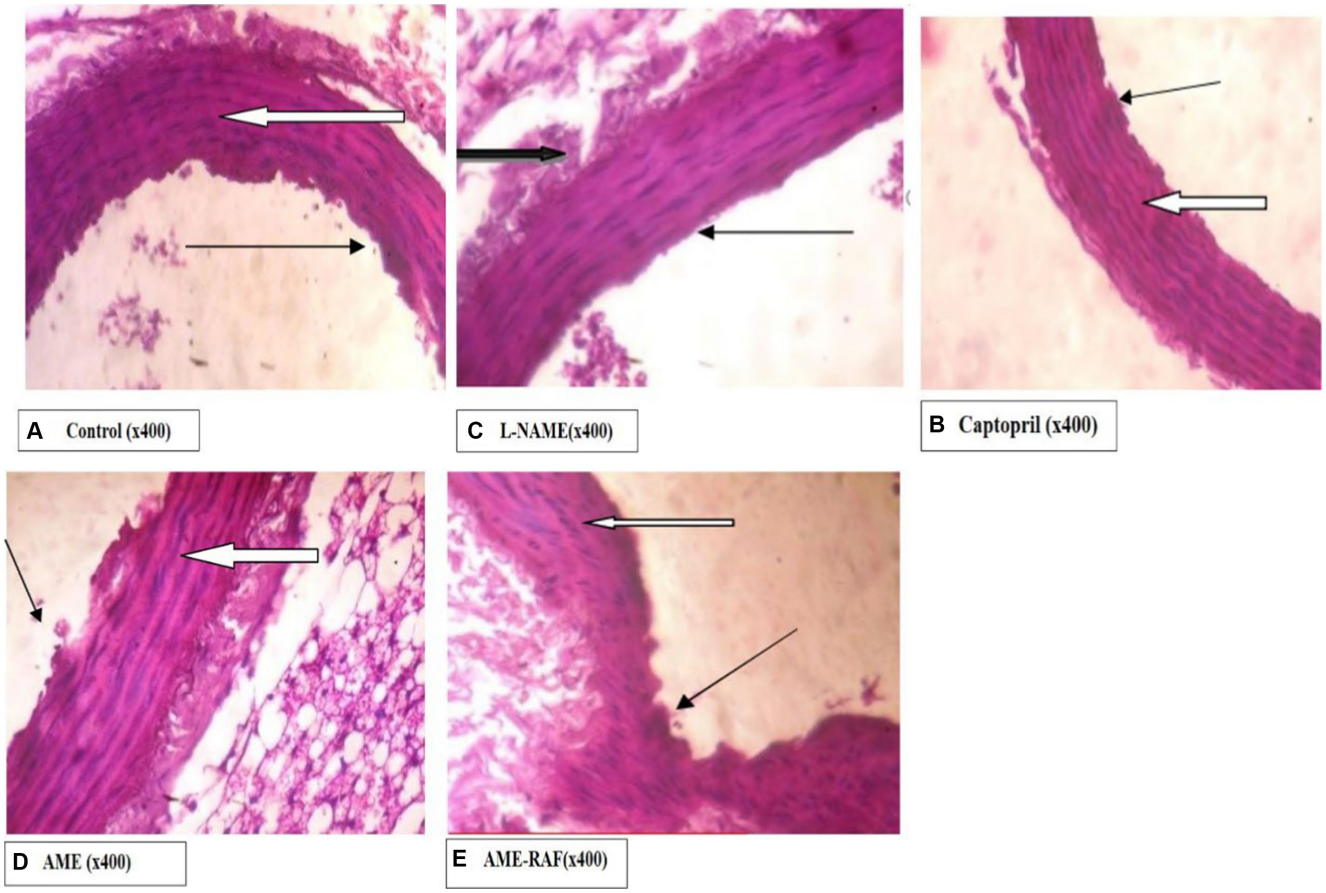
Photomicrograph of aorta sections. **(A)** Photomicrograph showing normal appearance. The aortic media appear normal, and there are many normal parallel lamellae (white arrow). *Tunica intima* appears normal (slender arrow). **(B)** Photomicrograph showing aortic media appearing normal. There are many normal parallel lamellae (white arrow). Tunica intima appears normal (slender arrow). The serosa layer shows moderate infiltration of inflammatory cells (black arrow). **(C)** Photomicrograph showing normal appearance, the aortic media appear normal. There are many normal parallel lamellae (white arrow). Tunica intima appears normal (slender arrow). **(D)** Photomicrograph showing normal appearance, the aortic media appear normal, and there are many normal parallel lamellae (white arrow). Tunica intima appears normal (slender arrow). A mildly infiltrated serosa layer is seen (black arrow). **(E)** Photomicrograph showing the normal appearance, the aortic media appear normal. There are many normal parallel lamellae (white arrow). Tunica intima seen show mild focal constriction (slender arrow).

Photomicrograph of the aorta sections is shown in Figure 10.

#### 3.6.2 Photomicrograph of the heart

**Figure 11:**
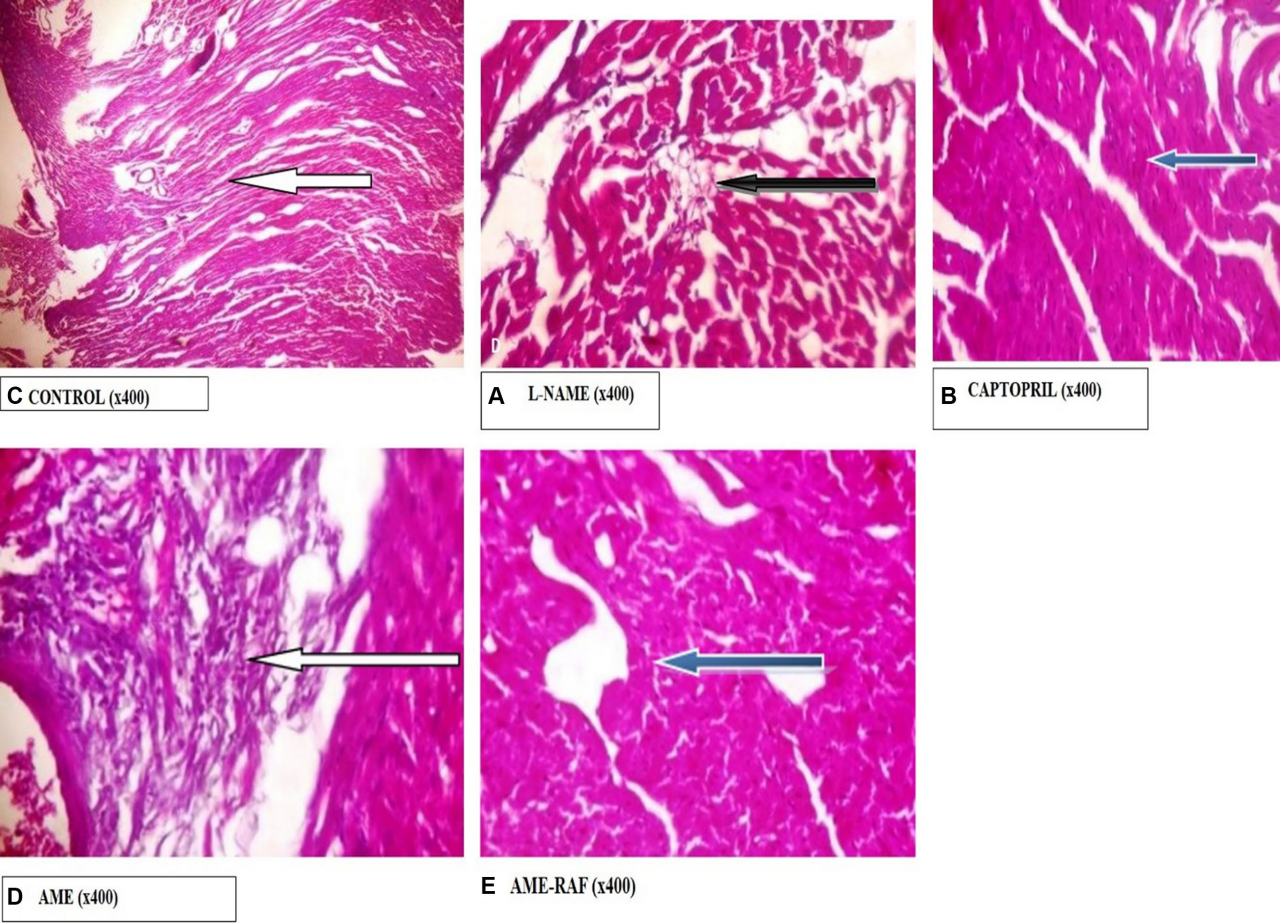
Photomicrograph of Heart sections. **(A)** Photomicrograph showing normal epicardial layer (white arrow) and normal myocardial layer. The myocytes are normal. **(B)** Photomicrograph showing the normal epicardial layer. Tunica intima seen also appear normal. The serosa layer shows mild infiltration of inflammatory cells (black arrow). **(C)** Photomicrograph showing normal epicardial layer and normal myocardial layer (blue arrow). The myocytes are also normal. **(D)** Photomicrograph showing area of moderate fibrosis (white arrow). **(E)** Photomicrograph showing normal epicardial and myocardial layers seen (blue arrow). The myocytes are also normal.

Photomicrograph of the heart sections is shown in Figure 11.

#### 3.6.3 Photomicrograph of the kidney

**Figure 12:**
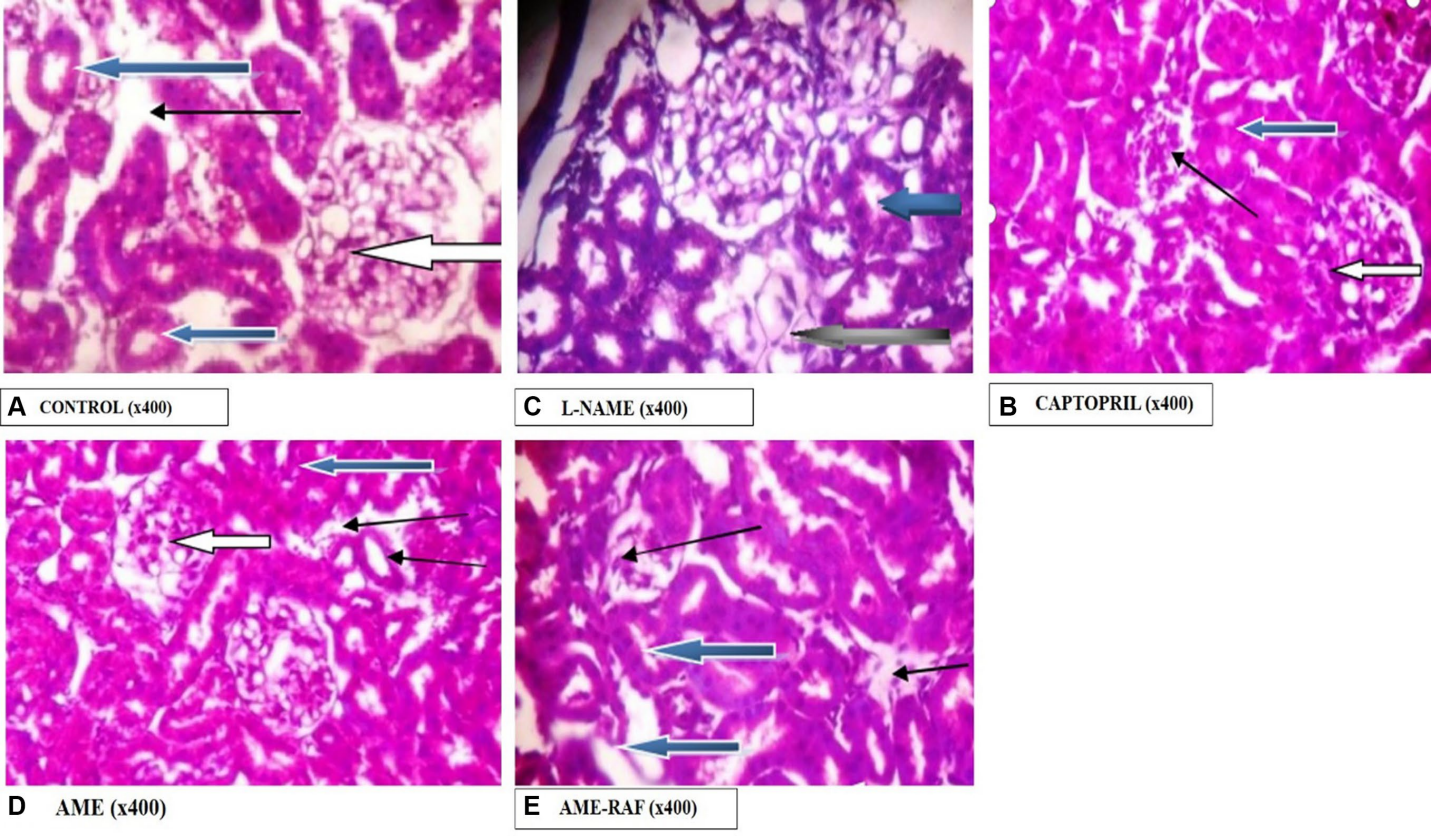
Photomicrograph of kidney sections. **(A)** Photomicrograph showing the renal cortex reveals normal glomeruli with normal mesangial cells and capsular spaces (white arrow), the renal tubules appear normal (blue arrow), and the interstitial spaces appear normal (slender arrow). **(B)** Photomicrographs showing the renal cortex. It shows normal glomeruli with normal mesangial cells and capsular spaces, the renal tubules appear normal (blue arrow), and the interstitial spaces appear mildly infiltrated by inflammatory cells. Few tubules are seen degenerated (black arrow). **(C)** Photomicrographs showing normal architecture. The renal cortex shows normal glomeruli with normal mesangial cells and capsular spaces (white arrow), the renal tubules appear normal (blue arrow), and the interstitial spaces show area of mild infiltration of inflammatory cells (slender arrow). **(D)** Photomicrographs showing the renal cortex which shows normal glomeruli with normal mesangial cells and capsular spaces (white arrow). The renal tubules appear normal (blue arrow), and the interstitial spaces show moderate vascular congestion (slender arrow). **(E)** Photomicrographs showing normal architecture as seen in higher magnification x400, the renal cortex showing normal glomeruli with normal mesangial cells and capsular spaces (white arrow). The renal tubules appear normal (blue arrow) and the interstitial spaces show scanty infiltration of inflammatory cells (slender arrow).

Photomicrograph of the kidney sections is shown in Figure 12.

## 4 Discussion

Hypertension over time has been referred to as the “silent killer” because there are no early warning signs or symptoms. It is a persistent rise in arterial blood pressure (25). Although the main reason or risk factor for most occurrences of hypertension is unknown, genetics, a high-salt diet, and psychological issues have all been connected to the condition (31). According to Adedapo et al. (32, 33), hypertension is the most prevalent non-communicable disease in Nigeria and its consequences cause about 25% of emergency room admissions in metropolitan hospitals.

The results demonstrated that *A. muricata* leaf extracts exhibited a protective effect against hypertension by modulating oxidative stress and reducing inflammation, as demonstrated by the alterations in inflammatory and antioxidant indicators in rats treated with *A. muricata* leaf extract after being given L-NAME-induced hypertension. The study demonstrated that L-NAME-induced hypertension was accompanied by increased systolic, diastolic, and mean arterial blood pressure, this finding is consistent with L-NAME’s non-specific inhibition of nitric oxide synthase, leading to hypertension (23). Treatment with *A. muricata* leaf extract significantly reduced the high blood pressure parameters, with the aqueous methanol extract at 200 mg/kg demonstrating a greater reduction than captopril, the conventional antihypertensive medication (40 mg/kg). This finding is consistent with previous research reporting a decrease in blood pressure in *A. muricata*-treated rats (34, 35). The blood pressure-lowering effect of *A. muricata* leaf extract observed in the present study may be attributed to the presence of polyphenols and flavonoids, which are frequently present in plant extracts and have been linked to hypotensive effects (36, 37). The crude extract of *A. muricata* contains a high level of phenols, which may be responsible for its anti-inflammatory and antioxidant effects. Since the majority of *A. muricata’s* phenolic compounds are soluble in water, they are regarded as the plant’s most important phytochemicals (18). Thirty-four phenolic compounds have been identified, from *A. muricata* leaves, including gallic acid, luteolin 3′7-di-O-glucoside, robinetin, taxifolin, and quercetin (38).

There was a significant decrease in the serum levels of triglyceride, total cholesterol, low-density lipoproteins (LDL), very low-density lipoproteins (VLDL), atherogenic index (AI), HDL/LDL ratio, and coronary risk index in the AME and RAF-treated rats. The HDL/LDL ratio of rats treated with AME and RAF showed a considerable improvement after receiving *A. muricata*. High plasma levels of LDL are indicated by a low HDL/LDL ratio, which may be proatherogenic (39). Furthermore, AME and RAF enhanced the HDL of the *A. muricata-*treated rats and reduced their atherogenic index to the level of control rats. A high atherogenic index typically indicates that vital organs like the heart, coronary vessels, aorta, liver, and kidneys may have foam cells, plaque, or fatty infiltration (40). The higher the atherogenic index, the more vulnerable are the above organs to oxidative injury (41). *A. muricata* reduced the atherogenic index and coronary risk index which are frequently regarded as better markers of the risk of coronary heart disease than individual lipoproteins (40). This suggests that *A. muricata* has atheroprotective properties, possibly due to part role of flavonoids and other phenolic compounds that is present in the leaf extract of the plant (42). The significant decrease in blood pressure, atherogenic risk index, and coronary risk index by captopril suggests that angiotensin-converting enzyme inhibitors can reduce blood pressure and hypercholesteremia concurrently. Given the strong relationship between AT1 receptor activation and blood pressure regulation, alterations in AT1 receptor properties may increase blood pressure. In cultured vascular smooth muscle cells and hypercholesterolemic rabbits, LDL elevates AT1 receptor expression (43). Nevertheless, despite evidence linking oxidized LDL to the development of atherosclerotic plaques, oxidized LDL, in contrast to natural LDL, did not affect AT1 receptor mRNA expression or angiotensin II-induced mitogenesis in vascular smooth muscle cells from rat thoracic aorta (43). This would imply that alterations in AT1 receptor expression brought on by LDL could subsequently act as a mediator for blood pressure rise and the onset of atherosclerosis.

Oxidative stress is known to be caused by the decreased activity of antioxidants and antioxidant enzymes, as well as increased levels of reactive oxygen species (ROS) or reactive nitrogen species (RNS), such as peroxynitrite. Increased activities of free radicals have been proposed to be a major pathway of hypertension (44). Although L-NAME can inhibit endothelial nitric oxide synthase to cause hypertension through increased peripheral resistance (45), it seems L-NAME can cause hypertension through other alternative pathways. We observed that a high level of aortic tissue nitrite correlates with enhanced production of MDA and decrease in antioxidant level of aortic tissue of non-treated hypertensive rats. After the inhibition of endothelial NOS by L-NAME, there is a possibility of enhanced inducible NOS activity in the aorta, leading to the production of abnormal NO, which is capable of reacting with oxygen-derived free radical superoxide anion. The NO was described to possess a double-edged sword effect by Mocellin et al. (46) because of its physiological (intracellular signaling mediator, neurotransmitter) and pathological roles. Interaction with oxygen-derived free radicals as a major pathway of hypertension as described by Gutowski and Kowalczy (47), can be linked to its role in pathological conditions. In addition, the superoxide anion effect on blood vessels has been linked to vasoconstriction, an abnormal change in endothelial function, and diminished NO bioavailability during hypertension (48). Also, the generation of ROS by NADPH oxidase and the stimulation of redox-dependent signaling cascades have been linked with angiotensin II-induced hypertension (48). In this study, the aorta, heart, and kidney of rats treated with L-NAME, AME, and RAF of *A. muricata* at higher doses demonstrated a considerable boost in the levels of catalase, GSH, SOD, and GST. The enhanced activities of antioxidants and enzymes might be responsible for the vasodilation and antihypertensive effect of *A. muricata*. The reduced level of antioxidants (GSH) and antioxidant enzymes (catalase, SOD, and GST) in the renal and myocardial tissue of L-NAME hypertensive rats along with increased serum levels of urea, further support the harmful effect of hypertension vital organs like the heart, and kidney. Also, rats given L-NAME had increased malondialdehyde (MDA) levels in their renal and myocardial tissue. An increase in malondialdehyde’s tissue level means lipid peroxidation by peroxynitrite; this is a perfect biomarker of oxidative stress (49). The study of Alabi et al. (50) suggests that peroxidation of membrane lipids due to excessive production of peroxynitrite can alter membrane permeability, and cause further liberation of cytokines, free radicals, and infiltration of tissues with leucocytes. The malondialdehyde levels of the aortic, renal, and cardiac tissue of rats treated with AME and RAF were significantly decreased, compared to the L-NAME-induced hypertension group. The activities of AME and RAF against lipid peroxidation in the treated rats support the important role of peroxynitrite and oxidative stress in the pathophysiology of hypertension and correlate with studies that showed that MDA level was low when hypertensive rats were treated with *Phragmanthera incana* leaf ethanol extract (25, 51).

The increased level of pro-inflammatory cytokines such as interleukin-1 beta and tumor necrosis factor-alpha (TNF-α) has made hypertension a low-grade inflammatory disease (IL-1β) (52, 53). In several models of hypertension, including the L-NAME model, there is proof that oxido-inflammatory processes and high blood pressure are related (54). Thus, determining the role of inflammation in hypertension requires examining the anti-inflammatory and antioxidant characteristics of traditional antihypertensive therapies. In the present study, L-NAME significantly elevated serum pro-inflammatory cytokine markers. Treatment of rats with *A. muricata* leaf extract reversed the elevated levels of serum IL-1β and TNF-α. Previous studies have demonstrated that the renin-angiotensin system (RAS)-mediated hypertension is abolished in rats lacking a functional gene for IL-1β (55). Moreover, a higher risk of myocardial infarction has been linked to an increase in IL-6 in hypertension (56, 57). Elevated TNF-α levels in renal or plasma tissues, in conjunction with concurrent oxidative stress development and elevated reactive oxygen species, may exacerbate endothelial dysfunction (57). These findings suggest that inhibiting inflammatory pathways could aid in the management of hypertension. Although several studies have connected the inflammasome and its byproducts to hypertension, more investigation is required to fully understand this relationship (10, 58). The anti-inflammatory properties of *A. muricata* leaves have also been documented in earlier research, underscoring the wide range of phytochemical composition and roles that these leaves possess (59, 60). *Annona muricata* leaves are a significant source of annonaceous acetogenins, a special class of long-chain fatty acid derivatives produced by the polypeptide pathway and belonging to the Annonaceae family (42). Acetogenins are the most predominant bioactive compounds of the Annonaceae family, with over 120 acetogenins reported from various parts of *A. muricata*, including the leaves, stems, bark, seeds, pulp, and fruit peel, and to date, approximately 46 acetogenins have been identified from the leaves including annomuricin A, annomuricin B, annomuricin C, muricatocin A, muricatocin B, and muricatocin C (60). The histology of the aorta, heart, and kidney showed the presence of inflammatory cells infiltrating tissues induced with hypertension during the study. However, treatment with captopril and *A. muricata* reversed this effect.

## 5 Conclusion

This study suggests the potential ameliorative effects of *Annona muricata* leaf extracts against L-NAME-induced hypertension in rats. Notably, the study showed the antioxidant and anti-inflammatory properties of *A. muricata* leaf extracts, which is seen in its ability to attenuate oxidative stress and inflammatory cytokines in L-NAME-induced hypertensive rats. Additionally, the study revealed the atheroprotective properties of *A. muricata* leaf extracts, as demonstrated by its capacity to lower coronary and atherogenic risk indices, and likely stop the onset of atherosclerosis, which is frequently caused by hypertension.

Further studies should be carried out to elucidate the underlying molecular mechanisms and signaling pathways involved in these protective effects.

## Data availability statement

The original contributions presented in the study are included in the article/supplementary material, further inquiries can be directed to the corresponding author.

## Ethics statement

The animal study was approved by University of Ibadan Ethical Approval Committee. The study was conducted in accordance with the local legislation and institutional requirements.

## Author contributions

ON: Conceptualization, Methodology, Writing – original draft, Writing – review & editing. BA: Methodology, Project administration, Writing – original draft, Writing – review & editing. JB: Conceptualization, Supervision, Validation, Writing – review & editing, Writing – original draft. FA: Data curation, Methodology, Writing – original draft, Writing – review & editing. O-AN: Software, Writing – original draft, Writing – review & editing. EI: Conceptualization, Supervision, Validation, Writing – review & editing, Writing – original draft.
